# Habitual Fish Oil Supplementation and Risk of Incident Inflammatory Bowel Diseases: A Prospective Population-Based Study

**DOI:** 10.3389/fnut.2022.905162

**Published:** 2022-07-12

**Authors:** Xiaoxu Huang, Yin Li, Pan Zhuang, Xiaohui Liu, Yu Zhang, Pianhong Zhang, Jingjing Jiao

**Affiliations:** ^1^Department of Clinical Nutrition, The Second Affiliated Hospital, Zhejiang University School of Medicine, Hangzhou, China; ^2^Department of Nutrition, School of Public Health, Department of Clinical Nutrition, The Second Affiliated Hospital, Zhejiang University School of Medicine, Hangzhou, China; ^3^Department of Food Science and Nutrition, Zhejiang Key Laboratory for Agro-Food Processing, Fuli Institute of Food Science, College of Biosystems Engineering and Food Science, Zhejiang University, Hangzhou, China

**Keywords:** Crohn’s disease, fish oil supplementation, inflammatory bowel diseases, ulcerative colitis, UK Biobank, C-reactive protein, album

## Abstract

**Background:**

Inflammatory Bowel Diseases (IBDs) have been emerging in recent years with the advance of global industrialization and diet pattern transformation. Marine n-3 polyunsaturated fatty acids (n-3 PUFAs), enriched in fish oils, have well-known human health promotion. Evidence on the association of fish oil supplementation with the risk of developing IBDs was scarce. This study aimed to examine the association between the use of fish oil supplements and the risk of developing inflammatory bowel diseases (IBDs) among the general population.

**Methods:**

We conducted a prospective cohort study of 447,890 participants aged 40–69 years from the UK Biobank. A touch screen questionnaire was used to get the data about fish oil intake at baseline. Incident diagnoses of IBDs were ascertained by the International Classification of Diseases (ICD-9 and ICD-10) or self-report. Cox proportional hazards model was applied to calculate hazard ratios (*HRs*) and 95% confidence intervals (*CIs*) of developing IBDs and their subtypes.

**Results:**

We documented 1,646 incident cases of IBDs, including 533 incident cases of Crohn’s disease (CD) and 1,185 incident cases of ulcerative colitis (UC) during an average of 8 years of follow-up. After multivariate adjustment, the use of fish oil was associated with a 12% lower risk of IBDs (*HR*: 0.88, 95% *CI*: 0.78–0.99, *p* = 0.03) compared with non-consumers. For subtypes of IBDs, fish oil supplementation was inversely associated with a 15% lower risk of UC (*HR*: 0.85, 95% *CI*: 0.75–0.99, *p* = 0.02) but was not correlated with the risk of CD (*p* = 0.22). Besides, fish oil supplementation showed a significant inverse correlation with baseline CRP levels (β = –0.021, *p* < 0.001) and a positive association with baseline albumin levels (β = 0.135, *p* < 0.001) after adjustment for multiple variates.

**Conclusion:**

Habitual intake of fish oil supplements was associated with a lower risk of IBDs and UC. Fish oil users tended to have lower baseline C-reactive protein levels and higher baseline albumin levels compared with non-users. It was concluded that fish oil supplement use may be recommended for the prevention and control of IBDs.

## Introduction

Inflammatory bowel diseases (IBDs) are non-specific chronic gastrointestinal tract inflammatory disorders, mainly including Crohn’s disease (CD) and ulcerative colitis (UC). The Global Burden of Diseases, Injuries, and Risk Factors Study reported that approximately 6.8 million people around the world had suffered from IBDs in 2017 (1–3). The population of IBDs has been rising in recent years with the advance of global industrialization and diet pattern transformation (3–5), which brings a heavy financial burden to patients’ families and society. It is urgent to formulate beneficial roles of social and dietary factors in controlling the prevalent trend of epidemic IBDs. Although the pathogenesis of IBDs is still unclear, genetic characteristics, environmental or microbial factors, and immune responses were involved in the etiology of IBDs (3). Diet has been reported to play an important role in IBDs by influencing the composition and functionality of the microbiome. Various dietary therapies have also become a potent tool for the remission of IBDs in some clinical studies (6). Marine n-3 polyunsaturated fatty acids (n-3 PUFAs), which are enriched in fish oils, have well-known anti-inflammatory, antiplatelet aggregatory, vasodilation, vasoconstriction, and ameliorating immune response effects for human health promotion (7–9). Interleukin-10 (IL-10) which is produced by resolvin E1 (RvE1) is regarded as the predominant anti-inflammatory cytokine in the intestine (10). RvE1, derived from n-3 PUFAs, also promotes intestinal mucosal repair (11). In addition, fish oil supplementation may weaken cellular immune responses, contributing to lower expression of Th17 cell type cytokine genes which play a part in the etiology of IBDs (12). Overall, n-3 PUFAs may be beneficial for preventing the occurrence of IBDs by regulating inflammatory mediators and ameliorating immune responses (13–17).

In humans, previous epidemiology studies tried to shed light on the association between dietary intake of n-3 PUFAs and the risk of IBDs, but the results were inconsistent. A case-control study found no significant association between dietary n-3 PUFA intake and the risk of UC in Caucasians (18), whereas a Japanese multicenter case-control study revealed that dietary n-3 fatty acid intake was correlated with the increase in CD risk (19). Another large prospective study reported that a long-term dietary intake of n-3 PUFAs was associated with a lower risk of UC in 170,805 women in the United States Nurses’ Health Study (20). Similarly, intake of docosahexaenoic acid (DHA), a major ingredient of marine n-3 PUFAs and fish oils, was inversely correlated with the incidence of IBDs in several studies (21–23). A meta-analysis of observational studies suggested no significant association between dietary n-3 PUFA intake and the risk of IBDs but indicated a significant inverse association for the risk of UC (pooled effect size: 0.75, 95% *CI*: 0.57–0.98, *p* = 0.03) (24). However, another meta-analysis did not observe an association between dietary fat intake including PUFA intake and the incidence of UC (18, 25).

Therefore, whether n-3 PUFA supplementation is beneficial for a lower incidence of IBDs and its subtypes remains controversial due to limited participants and IBDs cases in most studies. In the current study, we aim to conduct a large-scale population-based cohort study among participants aged 40–69 years and investigate the association between fish oil use and the incidence of IBDs and their subtypes in the UK Biobank.

## Materials and Methods

### Study Design and Participants

**FIGURE 1:**
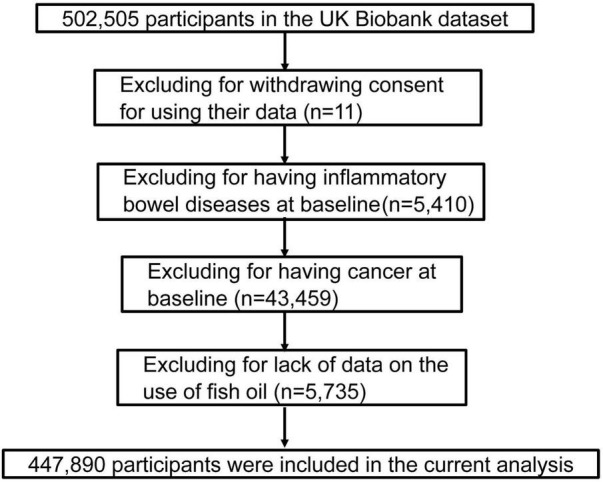
Flow chart of eligible participants in this study.

The UK Biobank is a prospective population-based cohort study that included a total of 502,505 participants, including 208,434 men and 239,456 women aged 40–69 years who were recruited between 2006 and 2017 in the United Kingdom (26). They were invited to complete a series of touch screen questionnaires, provide biological samples, and take various physical examinations at baseline. Informed consent was provided by each participant, and the UK Biobank’s study protocol was approved by the United Kingdom North West Multi-centre Research Ethics Committee. After excluding participants’ withdrawing on subsequent follow-up (*n* = 11), patients with IBDs (*n* = 5,410) and cancer (*n* = 43,459) at baseline, and those lacking data on the fish oil use (*n* = 5,735), the remaining 447,890 participants were included in the final analysis (Figure 1).

### Assessment of Fish Oil Use and Covariates

Participants were required to complete some questions from a series of touch screen questionnaires, including the question that “Do you regularly take any of the following?” in their own assessment centers and they could answer this question with a list of supplements including fish oils. To assess the reproducibility and effectiveness of the use of fish oil, two repeated surveys were conducted among 18,093 and 44,366 participants, respectively. In the first repeated survey completed from 2012 to 2013, 72.2% of fish oil consumers at baseline were reported to continue to use fish oil supplements (Spearman *r* = 0.61), while in the second repeated survey conducted in 2014 and later, 55.2% of fish oil users at baseline continued to take fish oil supplements (Spearman *r* = 0.47) (Supplementary Table 1).

Covariates included age, sex, race, assessment centers, body mass index (BMI), education, Townsend deprivation index (TDI) (27), household income, smoking status, alcohol consumption, physical activity, other dietary supplementation use, medication, and dietary intake. BMI was defined as the ratio of weight to squared height (kg/m^2^). The metabolic equivalent of task (MET) was computed according to the International Physical Activity Questionnaire short form (28). The use of aspirin, non-steroidal anti-inflammatory drugs (NSAIDs), and/or hormones was also assessed at baseline. Participants were required to answer 29 questions about the frequency of food intake and 18 questions on alcohol consumption by the touch screen questionnaires after the entry in the current study. We also referred to the definition that described the ideal intake of dietary components for cardiometabolic health and made a healthy diet score for each participant (Supplementary Table 2) (29). The healthy diet score was calculated by 10 kinds of dietary components (fruits, vegetables, whole grains, fish, dairy products, vegetable oil, refined grains, processed meat, unprocessed meat, and sugar-sweetened beverages) and each food had its own intake goal for keeping cardiometabolic health. Once the intake goal was met (Supplementary Table 2), one point was given for each favorable diet factor. The total healthy diet score ranged from 0 to 10. A healthier diet was related to a higher diet score.

The levels of C-reactive protein (CRP) and albumin at baseline were also measured (30). Due to the right-skewed distribution of CRP values, log (units + 1) of CRP values were used in our analyses (31). More detailed information about the study can be achieved online at https://biobank.ctsu.ox.ac.uk/showcase.

### Outcome Ascertainment

Definitions of IBDs are presented in Supplementary Table 3. A case was considered eligible if the participant had either a relevant inpatient International Classification of Diseases (ICD) code or self-reported illness (32). K51 and K50 were considered as the codes of UC and CD in ICD version 10, respectively. Alternatively, codes 556 and 555 could also be used for the judgment of UC and CD in ICD version 9, respectively. Self-reported IBD cases were documented during the assessment interview. If a participant had recorded diagnoses for both UC and CD, then the individual was only defined as one case of IBDs.

### Statistical Analysis

The follow-up duration of participants was from the date of attending baseline assessment until the time of IBD diagnosis, lost to follow-up, death, or the end of follow-up (March 31, 2017), whichever came first. We used Cox proportional hazards models to estimate the hazard ratios (*HRs*) and 95% confidence intervals (*CIs*) of IBDs in accordance with the use or non-use of fish oil supplements after adjustment for potential confounders in a group of stepwise covariate-adjusted models. Model 1 was adjusted for age, sex, race, assessment centers, BMI, education, TDI, household income, smoking status, alcohol consumption, physical activity, vitamin supplement use, mineral supplement use, aspirin use, hormone use, and NSAID drug use. Model 2 was additionally adjusted for the intake of oily fish, processed red meat, vegetables, fruits, whole grains, and cheeses. Model 3 was further adjusted for a healthy diet score based on model 1 given the potential interactions between different dietary components. We also assessed the associations of fish oil supplementation with baseline albumin levels and CRP levels in secondary analyses. Then, we performed subgroup analyses to evaluate the associations stratified by potential effect modifiers, including age, BMI, TDI, smoking status, alcohol consumption, physical activity, healthy diet score, healthy lifestyle score, vitamin supplement use, mineral supplement use, aspirin use, NSAID use, oily fish intake, and non-oily fish intake. The *p*-value for interaction was reckoned by adding the cross-product term of fish oil use with either of the above stratifying variables into the model. Moreover, an overall healthy lifestyle score based on BMI (<30 kg/m^2^), smoking (never), physical activity (≥600 MET min/week), and healthy diet (yes) were also considered in subgroup analyses (33). When participants satisfied any of the scoring criteria, they could get one point. The healthy lifestyle score was the sum of five component scores and ranged from 0 to 5. Sensitivity analyses were conducted by further adjusting for coffee intake, contraceptive use, depression, or further excluding extreme BMI values. Besides, we further adjusted CRP and albumin levels to test whether the association was weakened. Participants were grouped into three categories according to CRP concentration (<5 mg/l, 5–10 mg/l, ≥10 mg/l) (34). For albumin, those with 35–50 g/L albumin were seen as the reference group and the rest of the participants were classified into two categories (<35 g/l, ≥50 g/l) (35). To minimize the possibility of reverse causation, we also further excluded incident IBD cases that occurred within 2 years in a sensitivity analysis. All the statistical analyses were conducted by SAS version 9.4 (SAS Institute, Cary, NC, United States). Two-sided *p*-values less than 0.05 indicated statistical significance.

## Results

### Baseline Characteristics

Our study followed 447,890 participants with 3,606,243 person-years in the UK Biobank study. During an average of 8 years of follow-up, we confirmed 1,646 IBD cases (incidence 46 per 100,000 person-years), including 533 CD cases (incidence 15 per 100,000 person-years) and 1,185 UC cases (incidence 33 per 100,000 person-years). Table 1 shows the baseline characteristics of participants according to whether they used fish oil supplements. Compared with non-users, fish oil users were generally older and more often female, and had lower household income and TDI. They also tended to be more physically active, not current smokers, have lower BMI, and were more likely to drink alcohol and use aspirin, NSAID drugs, vitamins, and minerals. They preferred oily fish, non-oily fish, vegetables, fruits, and whole grains, whereas they were less likely to consume processed meat, refined grains, cheeses, and sugar-sweetened beverages (SSB). Moreover, the dietary pattern of fish oil users was healthier than non-users.

**TABLE 1:** Basic characteristics of participants by use of fish oil in the UK Biobank cohort.

Characteristics	Overall	Fish oil non-users	Fish oil users
	(*n* = 447,890)	(*n* = 308,111)	(*n* = 139,779)
Male, %	46.5	47.6	44.2
Age, years	56.2 (8.1)	55.2 (8.2)	58.4 (7.5)
**Race, %**			
White	94.1	93.8	94.7
Asian	2.4	2.6	1.8
Black	1.7	1.6	1.8
Mixed	0.6	0.6	0.6
Others	0.9	1.0	0.8
BMI, kg/m^2^	27.4 (4.8)	27.5 (4.9)	27.2 (4.6)
**Household income (£), %**			
<18,000^a^	19.0	18.5	20.3
18,000 to 30,999	21.6	20.6	23.8
31,000 to 51,999	22.6	23.0	21.7
52,000 to 100,000	17.8	19.0	15.1
>100,000	4.7	5.3	3.6
Townsend deprivation index	−1.3 (3.1)	−1.2 (3.1)	−1.5 (3.0)
**Education, %**			
College or University degree	32.6	33.7	30.2
Vocational qualifications	11.7	11.3	12.6
Optional national exams at ages 17–18 years	26.7	26.7	26.7
National exams at age 16 years	16.8	16.1	18.3
Others	1.0	1.0	1.1
Physical activity, MET-h/wk	44.3 (45.3)	42.7 (44.8)	47.8 (46.4)
**Smoking status, %**			
Never	55.0	55.5	54.1
Previous	34.0	32.5	37.3
Current	10.6	11.6	8.2
**Alcohol consumption, %**			
Never or special occasions only	19.3	19.8	18.3
1 to 3 times/month	11.2	11.4	10.7
1 or 2 times/week	25.9	25.8	26.0
3 or 4 times/week	23.2	22.9	24.1
Daily or almost daily	20.3	20.1	20.8
NSAIDs use, %	37.6	36.1	41.1
Aspirin use, %	13.9	12.7	16.5
Hormone, %	3.9	3.6	4.6
Vitamin supplementation, %	31.4	20.1	56.3
Mineral supplementation, %	12.1	8.1	20.9
**Dietary consumption, %**			
**Oily fish, times/week**			
<1	44.2	47.9	36.1
1	37.4	35.8	41.1
≥2	17.8	15.7	22.4
**Non-oily fish, times/week**			
<1	33.8	35.8	29.4
1	49.4	48.2	52.2
≥2	16.2	15.4	18.0
**Poultry, times/week**			
<2	51.6	51.7	51.4
2–4	45.9	45.7	46.2
>4	2.3	2.4	2.2
**Processed meat, times/week**			
<1	39.5	38.5	41.8
1	29.1	28.9	29.6
≥2	31.2	32.4	28.5
**Unprocessed red meat, times/week**			
<2.0	49.8	49.6	50.2
2.0–4.0	41.8	41.8	41.9
>4.0	8.2	8.4	7.8
**Vegetable, servings/day**			
<1.0	18.0	19.6	14.6
1.0–2.9	72.6	71.4	75.3
≥3.0	8.7	8.3	9.5
**Fruit, servings/day**			
<2.0	35.2	38.5	27.9
2.0–3.9	47.5	45.8	51.1
≥4.0	17.1	15.4	20.8
**Whole grain, servings/day**			
<1.0	42.1	45.2	35.3
1.0–2.9	43.4	41.0	48.8
≥3.0	13.3	12.6	14.9
**Refined grain, servings/day**			
<1.0	58.2	55.9	63.3
1.0–2.9	31.3	32.8	28.1
≥3.0	9.3	10.1	7.6
**Cheese, times/week**			
<2	40.5	39.9	41.8
2–4	44.1	44.2	43.9
>4	12.9	13.5	11.5
**Coffee, cups/day**			
<1	29.3	29.7	28.5
1–2	38.6	37.0	42.1
≥3	31.8	33.0	29.2
Sugar-sweetened beverages consumer, %	81.7	83.0	78.8
Healthy diet score	3.0 (1.4)	2.9 (1.4)	3.3 (1.4)

*BMI, body mass index. Values are means (SD) or percentages unless stated otherwise. ^a^£1.00 = $1.30, €1.20. NSAIDs, non-steroidal anti-inflammatory drugs. Hormone, hormone replacement therapy.*

### Fish Oil Supplementation and Incident Inflammatory Bowel Disease Risk

In our age- and sex-adjusted model, we did not observe any significant association between fish oil use and the risk of incident IBDs (Table 2). After adjustment for other demographic characteristics and the use of other supplements and medications (model 1), the use of fish oil supplements was inversely associated with a 13% lower risk of IBDs (*HR*: 0.87, 95% *CI*: 0.78–0.98; *p* = 0.02). The results also did not remarkably change after further adjustment for potentially related dietary factors, such as oily fish, processed red meat, vegetables, fruits, whole grains, and cheeses (model 2). Finally, fish oil use was also correlated with the risk of IBDs after adjustment for a healthy diet score (model 3). The *HR* (95% *CI*) of IBD risk associated with fish oil use was 0.88 (0.78–0.99) (*p* = 0.03) in this model. Then, we separately analyzed the associations with the risk of UC and CD and found different results. The fish oil intake was inversely associated with a 15% lower risk of UC (*HR*: 0.85, 95% *CI*: 0.75–0.99, *p* = 0.02) (Table 3) after the adjustment for multiple variates, but was not significantly associated with the risk of the CD (*p* = 0.22).

**TABLE 2:** HRs (95% CIs) of inflammatory bowel diseases according to fish oil use in the UK Biobank.

	Fish oil non-users	Fish oil users	*P*-value
	(*n* = 308,111)	(*n* = 139,779)	
Number of cases	1,143	503	
Person-years	2,477,757	1,128,486	
Age- and sex-adjusted HR (95% CI)	1 [Ref]	0.93 (0.83–1.03)	0.16
Model 1^a^	1 [Ref]	0.87 (0.78–0.98)	**0.02**
Model 2^b^	1 [Ref]	0.88 (0.79–0.99)	**0.04**
Model 3^c^	1 [Ref]	0.88 (0.78–0.99)	**0.03**

*CI, confidence interval; HR, hazard ratio. ^a^Adjusted for age, sex, race (White, Asian, Black, mixed, or other ethnic group), assessment centers (22 categories), BMI (in kg/m^2^; <18.5, 18.5 to 25, 25 to 30, 30 to 35, ≥35, or missing), education (college or university degree, vocational qualifications, optional national exams at ages 17–18 years, national exams at age 16 years, others, or missing), Townsend deprivation index (quintiles), household income (<£18,000, £18,000-£30,999, £31,000-£51,999, £52,000-£100,000, >£100,000, or missing), smoking status (never, former, current, or missing), alcohol consumption (never, special occasions only, 1–3 times/month, 1 or 2 times/week, 3 or 4 times/week, or daily/almost daily), physical activity (in MET-h/wk; quintiles), vitamin supplement use (yes or no), mineral supplement use (yes or no), aspirin use (yes or no), hormone use (yes or no), and non-steroidal anti-inflammatory drugs use (yes or no). ^b^Additionally adjusted for oily fish (<1, 1, or ≥2 times/week), processed red meat (<1, 1, or ≥2 times/week), vegetables (<1, 1–3, or ≥3 servings/day), fruits (<2.0, 2.0–3.9, ≥4.0 servings/day), whole grains (<1.0, 1.0–2.9, ≥3.0 servings/day), and cheeses (<2, 2–4, >4 times/week) based on model 1. ^c^Further adjusted for healthy diet score (quintiles) based on model 1. Bold value indicates statistical significance.*

**TABLE 3:** HRs (95% CIs) of ulcerative colitis and Crohn’s disease according to fish oil use in the UK Biobank.

	Fish oil non-users	Fish oil users	*P*-value
	(*n* = 308,111)	(*n* = 139,779)	
**Ulcerative colitis**			
Number of cases	826	359	
Person-years	2,478,885	1,128,999	
Age- and sex-adjusted HR (95% CI)	1 [Ref]	0.91 (0.81–1.04)	0.16
MV-adjusted HR (95% CI)^a^	1 [Ref]	0.85 (0.75–0.99)	**0.02**
**Crohn’s disease**			
Number of cases	376	157	
Person-years	2,480,599	1,129,753	
Age- and sex-adjusted HR (95% CI)	1 [Ref]	0.88 (0.73–1.06)	0.19
MV-adjusted HR (95% CI)^a^	1 [Ref]	0.88 (0.72–1.08)	0.22

*CI, confidence interval; HR, hazard ratio; MV, multivariable. ^a^MV-adjusted model was adjusted for age, sex, race (White, Asian, Black, mixed, or other ethnic group), assessment centers (22 categories), BMI (in kg/m^2^; <18.5, 18.5 to 25, 25 to 30, 30 to 35, ≥35, or missing), education (college or university degree, vocational qualifications, optional national exams at ages 17–18 years, national exams at age 16 years, others, or missing), Townsend deprivation index (quintiles), household income (<£18,000, £18,000-£30,999, £31,000-£51,999, £52,000-£100,000, >£100,000, or missing), smoking status (never, former, current, or missing), alcohol consumption (never, special occasions only, 1–3 times/month, 1 or 2 times/week, 3 or 4 times/week, or daily/almost daily), physical activity (in MET-h/wk; quintiles), vitamin supplement use (yes or no), mineral supplement use (yes or no), aspirin use (yes or no), hormone use (yes or no), non-steroidal anti-inflammatory drugs use (yes or no), and healthy diet score (quintiles). Bold value indicates statistical significance.*

### The Correlation Between Fish Oil Supplementation and Blood Biomarkers

At baseline, fish oil supplementation showed a significant inverse correlation with CRP levels (β = –0.021, *p* < 0.001) (Table 4) and a positive association with albumin levels (β = 0.135, *p* < 0.001) after adjustment for multiple variates.

**TABLE 4:** β coefficients of fish oil use for blood indicators from general linear regression analysis in the UK Biobank.

	β	SE	*P*-value
Albumin	0.135	0.010	**<0.001**
CRP^a^	−0.021	0.002	**<0.001**

*^a^Log (units + 1) of CRP values were used. Model was adjusted for age, sex, race, assessment centers, BMI, education, Townsend deprivation index, household income, smoking status, alcohol consumption, physical activity, vitamin supplement use, mineral supplement use, aspirin use, hormone use, non-steroidal anti-inflammatory drugs use, and healthy diet score. Bold value indicates statistical significance.*

### Subgroup and Sensitivity Analyses

**FIGURE 2:**
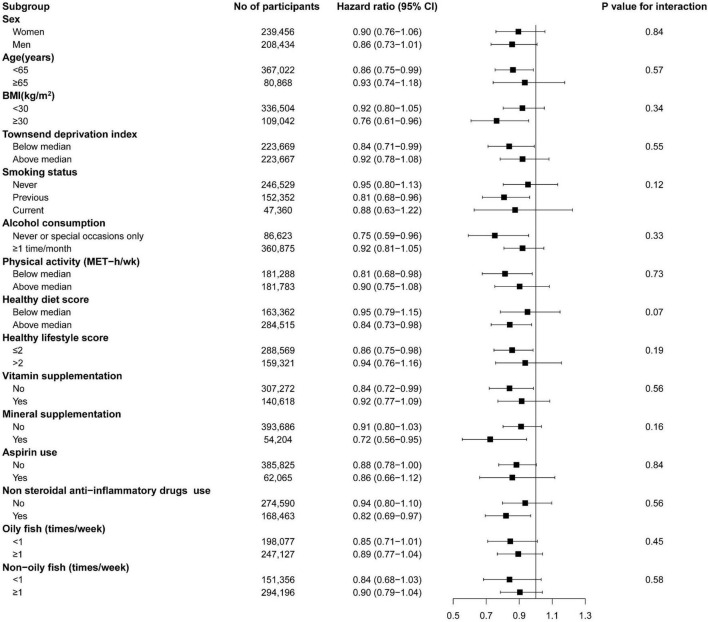
The association of fish oil supplementation with inflammatory bowel disease risk in subgroup analyses. BMI, body mass index; MET, metabolic equivalent of task. Forest plots show the multivariable HRs of IBDs associated with fish oil use in subgroups. Model was adjusted for age, sex, race, assessment centers, BMI, education, Townsend deprivation index, household income, smoking status, alcohol consumption, physical activity, vitamin supplement use, mineral supplement aspirin use, hormone use, non-steroidal anti-inflammatory drugs use, and healthy diet score. Horizontal line represents 95% CIs.

We did not find a significant interaction between fish oil use and risk of all-cause IBDs, when the analyses were stratified by sex, age, BMI, TDI, smoking, alcohol consumption, physical activity, healthy diet score, healthy lifestyle score, vitamin supplement use, mineral supplement use, aspirin use, NSAID drug use, oily fish intake, or non-oily fish intake (Figure 2). Sensitivity analyses showed that the documented significant association between fish oil supplementation and the incidence of IBDs did not change substantially after further adjustment for coffee intake, depression, and contraceptive use (Supplementary Table 4). The relationship was still significant after further adjustment for CRP (*HR*: 0.89, 95% *CI*: 0.79–0.99, *p* = 0.04) and albumin (*HR*: 0.88, 95% *CI*: 0.79–0.99, *p* = 0.03). Meanwhile, the association was also robust after excluding the incident IBD cases that occurred within 2 years or participants who had extreme BMI values (<18.5 or > 40 kg/m^2^).

## Discussion

In this study, we found that habitual fish oil use was correlated with a 12% lower risk of developing IBDs after adjustment for potential confounders. For IBD subtypes, fish oil supplementation was significantly associated with a lower risk of UC but not CD. We also assessed inverse associations of fish oil supplementation with baseline albumin levels and CRP levels.

To our knowledge, our study is the most extensive prospective cohort study to investigate the association between habitual marine n-3 PUFA intake and the risk of incident IBDs. We showed that the incidence of UC (33 per 100,000 person-years) and CD (15 per 100,000 person-years) was both higher than those that were previously reported in Europe (36). The increased incidence of UC and CD in the current study was in line with the elevating global trend (1). Some pieces of evidence from the previous epidemiological studies have supported our findings. Two case-control studies acknowledged that fish consumption had a protective role in the incidence of IBDs (37, 38). A nested case-control study in the European Prospective Investigation into Cancer and Nutrition (EPIC) agreed that a higher intake of n-3 PUFAs and DHA from the diet could be beneficial for a lower risk of developing UC (39). A United Kingdom prospective cohort study found similar results and reported a significant association of DHA intake with UC and a similar borderline significant association for total n-3 PUFA and eicosapentaenoic acid (EPA) intake (40). A meta-analysis including 282,610 participants and 2,002 IBD cases also observed that dietary long-chain n-3 PUFA intake was associated with a lower risk of UC (pooled effect size: 0.75, 95% *CI*: 0.57–0.98, *p* = 0.03) (24). However, fish consumption showed no significant association with UC and CD in two case-control studies (41, 42).

Dietary n-3 PUFA intake may be beneficial to the control of UC, but the inverse association was not significant in most studies due to other confounding dietary factors. Higher dietary intake of long-chain n-3 PUFAs was associated with a trend toward a 27% lower risk of developing UC in the EPIC cohort (20). Another meta-analysis revealed that the intake of n-3 PUFAs was not associated with the risk of UC, and only DHA showed a potential protective effect in the development of UC (25). The intake of n-3 PUFAs from the diet may be influenced by other food nutrients including fatty acids, such as n-6 PUFAs. Data from a European perspective cohort study supported that linoleic acid intake was positively correlated with the risk of UC (39), indicating the protective role of n-3 PUFAs from the diet may be weakened by other types of dietary fatty acids. Most studies reported that the intake of DHA played an important role in the development of UC (25, 39, 40). Given high amounts of marine n-3 PUFAs, the habitual use of fish oil supplements may elevate the daily intake level of n-3 PUFAs from the diet, which contributed to the inverse association with the occurrence of IBDs and UC. However, current research on the relationship between the use of fish oil supplements and the incidence of IBDs is still scarce. On the other hand, the outcomes from existing studies regarding dietary n-3 PUFA intake remain inconsistent due to small-scale participants, a small number of cases, and heterogeneity of diet assessment. In the current study, we found significant inverse associations and filled the gap of direct evidence on the protective role of fish oil supplementation in the risk of all-cause IBDs and UC since fish oil supplements contain much higher amounts of marine n-3 PUFAs than the dietary intake level of n-3 PUFAs from foods.

For another subtype of IBDs, conflicting results also existed for the association between n-3 PUFA intake and the risk of CD. A multicenter case-control study in Japan found a positive association between dietary n-3 PUFA intake and CD risk (19), whereas another Canadian population-based case-control study demonstrated an inverse association (43).

A recent meta-analysis revealed that dietary long-chain n-3 PUFA intake was not correlated with incident CD (pooled effect size in fixed model: 0.85, 95% *CI*: 0.59–1.23, *p* = 0.37) (24). Similarly, we found no association between fish oil use and incident CD in the current study. Although UC and CD present similar clinical symptoms, their pathogenesis and the course of developing diseases are obviously different (44), resulting in their possible different responses to fish oil supplementation. A further organ culture experiment investigated differential effects of fish oil-enriched enteral diet on UC and CD in tissue by showing an increase in IL-1ra/IL-1β cytokine ratios and thus improving inflammatory status, and proved that this modification effect was significantly more marked in UC compared with CD (45). The above study provided insights into the current finding that fish oil supplementation was conducive to the prevention of UC rather than CD.

The finding that fish oil use was correlated with a lower risk of IBDs in our study was supported by several plausible biological mechanisms. EPA as one of the key ingredients in fish oil is metabolized to prostaglandin E3 and leukotriene B5, which have anti-inflammatory properties (46). Besides, n-3 PUFAs can promote the release of phospholipases D from cell membranes and thus activate its anti-proliferative effects in lymphoid cells (47). The n-3 PUFAs also can decrease levels of secondary messengers involved in inflammation by inhibiting protein kinase C (PKC) activity (48). In addition, fish oil supplements may suppress cell-mediated immune responses by diminishing the expression of major histocompatibility complex class II molecules in peripheral blood monocytes (49). Furthermore, n-3 PUFAs reduced IL-1β and IL-18 gene expression in human adipocytes and macrophages (50). By upregulating gene expression of peroxisome proliferator-activated receptors, marine n-3 PUFAs could inhibit the activation of nuclear factor κB, which plays a key role in initiating genes encoding for inflammatory factors involving tumor necrosis factor-α (TNF-α) and Interleukin-6 (IL-6) (12). Last, n-3 PUFAs ameliorated gut microbiota composition and influenced the gut–brain axis, which increased the production of anti-inflammatory compounds, such as short-chain fatty acids, for the improvement of IBDs (15). Therefore, the interaction between n-3 PUFAs, immunity, and gut microbiota could be in favor of maintaining the intestinal wall integrity and communicating with host immune cells and alleviating the pathogenesis of IBDs. On the other hand, serum levels of CRP and albumin were all closely related to the occurrence of IBDs and are considered to be possible biomarkers for auxiliary diagnosis (30). Meta-analyses concluded that the intake of n-3 PUFA could significantly reduce the levels of inflammation markers, such as CRP, IL-6, and TNF-α (51), and elevated the serum level of albumin (52). Consistently, we showed that fish oil users had significantly lower levels of CRP and higher serum levels of albumin, which contributed to a lower risk of IBDs. Besides, a slight association was found after further adjustment for CRP, which may be explained that n-3 PUFA supplementation could reduce the risk of IBDs by alleviating inflammatory response.

There are several advantages. The current study investigated the associations with large numbers of participants and cases and the long duration of an average of 8-year follow-up using a prospective design, which maximally reduces the possibility of reverse causality and allows us to further enable the IBD subtypes and various subgroup analyses. Besides, we focused on the incident IBDs associated with the habitual use of fish oil supplements, indicating a purer and higher intake of n-3 PUFAs than dietary n-3 PUFA intake, which also fills the gap of n-3 PUFA supplementation in relation to the risk of IBDs. Additionally, we have a wealth of information on socioeconomic characteristics, lifestyle and dietary factors, and other covariates, which largely eliminated the interference from confounding factors. Sensitivity analyses also demonstrated the robustness of our findings.

Several limitations should be noted in this study. First, we lacked detailed information about fish oil use, such as intake dosage, intake duration, and the ratio of EPA to DHA in fish oils, which might help us deeply analyze the associations, such as dose–response analysis and the long-term role of supplementation in the risk of IBDs. Although regularly taking fish oil supplements was registered, the frequency of fish oil use could not be acquired. Nonetheless, we conducted repeated surveys to assess the reproducibility and validity of fish oil use and found that more than half of fish oil users at baseline continuously intake the supplements all the time. Second, we only measured CRP and albumin levels at baseline, which might not reflect a long-term inflammation status among participants. Thus, our findings could not reveal the causal effect of fish oil use on the levels of CRP and albumin. Third, our association outcomes might be biased by residual confounding that was unable to be corrected, such as the family history of IBDs. Finally, due to the nature of the observational study design, the causal relationship between fish oil use and the risk of IBDs could not be established.

## Conclusion

In conclusion, the habitual use of fish oil supplements was significantly associated with a lower incidence of IBDs and its subtype UC but not CD, which might partly be ascribed to the anti-inflammatory effect of marine n-3 PUFAs. Further studies should understand the pathogenesis of IBDs and the role of inflammatory improvement by various dietary components or supplements in the treatment of IBDs. Meanwhile, more trials with a large sample size and a long duration of follow-up are warranted to formulate the recommendations about intake of n-3 PUFA supplements to prevent the incident IBDs in the future.

## Data Availability Statement

The datasets presented in this article are not readily available because the data that support the findings of this study are available from UK Biobank project site, subject to successful registration and application process. Requests to access the datasets should be directed to https://biobank.ctsu.ox.ac.uk/.

## Ethics Statement

The studies involving human participants were reviewed and approved by the United Kingdom North West Multi-centre Research Ethics Committee. The patients/participants provided their written informed consent to participate in this study.

## Author Contributions

JJ and PiZ conceived and designed the study and the guarantors and responsible for the authenticity and accuracy of the study. XH, YL, PaZ, and XL cleaned, analyzed, and interpreted the data. XH and YL completed the manuscript. PaZ and XL provided expertise and assistance with the statistic. YZ, PaZ, and XL revised the manuscript. All authors finally approved the draft, contributed to the article, and approved the submitted version.

## Conflict of Interest

The authors declare that the research was conducted in the absence of any commercial or financial relationships that could be construed as a potential conflict of interest.

## Publisher’s Note

All claims expressed in this article are solely those of the authors and do not necessarily represent those of their affiliated organizations, or those of the publisher, the editors and the reviewers. Any product that may be evaluated in this article, or claim that may be made by its manufacturer, is not guaranteed or endorsed by the publisher.
