# Supplementary material for: Habitual Fish Oil Supplementation and Risk of Incident Inflammatory Bowel Diseases: A Prospective Population-Based Study
Source: Front Nutr. 2022 Jul 12;9:905162. doi: 10.3389/fnut.2022.905162 (PMC9315369; doi:10.3389/fnut.2022.905162)
Supplement: Supplementary file 1 [file Data_Sheet_1.docx]

*Supplementary Material*

**Supplementary methods**

**Dietary variables**

**Fruits**

Participants needed to answer the amounts of fruit intake per day. Besides directly answering the pieces of fruit intake, they could select “less than one”, “did not know”, and “prefer not to answer”. A serving was defined as a piece of fresh fruit or a five-piece of dried fruit.

**Vegetable**

Participants were asked “On average how many heaped tablespoons of vegetables would you eat per day?” The total tablespoons of cooked vegetables and salad or raw vegetables were calculated and three tablespoons were regarded as one serving.

**Refined grains and whole grains**

White, brown, or other non-whole grain bread and Biscuit cereal or other non-wholegrain cereal were all regarded as refined grains. The amount of whole grains intake was estimated across wholegrain bread intake and wholegrain cereal intake. One slice of bread or one bottle of cereal was deemed as one serving.

**Fish, cheese, and meat**

For oily fish, nonoily fish, cheese, processed meats, poultry, beef, lamb, and pork, participants were asked about the frequency of each item. They could select ”never“，” less than once a week“，”once a week“，”2-4 times a week“，”5-6 times a week“，” once or more daily“, ”do not know“，and ” prefer not to answer“. If participants drank milk and selected the type of milk, we assumed that they had one glass of milk per day. The total frequencies of unprocessed meats intake were summed up in the times of poultry intake, beef intake, lamb intake, and pork intake. The total consumption of dairy products was calculated by summing the cheese intake and the milk intake.

**Vegetable oil**

If participants mainly used flora pro-active/benecol spread and selected flora pro-active or Benecol, soft (tub) margarine, olive oil-based spread, polyunsaturated/sunflower oil-based spread, other low or reduced-fat spread, the vegetable oil was consumed. We assumed that the two slices of bread represent one serving of vegetable oil.

**Sugar-sweetened beverages**

Participants were requested to answer “which of the following do you never eat?”. If they selected “sugar or foods/drinks containing sugar”, the intake of sugar-sweetened beverages was deemed as “never”.

**Other variables**

Age and sex were treated as continuous variables. We grouped participants into 6 categories according to body mass index, including <18.5, 18.5 to 25, 25 to 30, 30 to 35, ≥35, or missing. Participants were asked about smoking status and could select “never”, “previous”, “current”, and “prefer not to answer”. Participants were divided into 4 categories, including non-smokers, past smokers, current smokers, or missing. Participants were grouped into 6 categories according to alcohol consumption (never, special occasions only, 1-3 times/month, 1 or 2 times/week, 3 or 4 times/week, or daily/almost daily). Race (white, Asian, black, mixed, or other ethnic groups), assessment center (22 categories), education (college or university degree, vocational qualifications, optional national exams at ages 17–18 years, national exams at age 16 years, others, or missing), Townsend deprivation index (quintiles), household income (<£18,000, £18,000-£30,999, £31,000-£51,999, £52,000-£100,000, >£100,000, or missing), physical activity (in MET-h/wk; quintiles), vitamin supplement use (yes or no), mineral supplement use (yes or no), aspirin use (yes or no), hormone use (yes or no), and nonsteroidal anti-inflammatory drugs use (yes or no) were adjusted in the model. We made additional adjustment for oily fish (<1, 1, or ≥2 times/week), processed red meat (<1, 1, or ≥2 times/week), vegetables (<1, 1-3, or ≥3 servings/day), fruits (<2.0, 2.0-3.9, ≥4.0 servings/day), whole grains (<1.0, 1.0-2.9, ≥3.0 servings/day), and cheeses (<2, 2-4, >4 times/week) in model 2. In final model, healthy diet score was adjusted as quintiles variables.

**Table of Contents**

**Table S1** Definitions of incident inflammatory bowel diseases

**Table S2** Diet component definitions used in the UK Biobank study

**Table S3** Spearman correlations between the fish oil use assessed at baseline and in resurveys

**Table S4** HRs (95% CIs) of inflammatory bowel diseases in sensitivity analysis

| **Supplementary Table 1. Definitions of incident inflammatory bowel diseases** | | | |
| --- | --- | --- | --- |
|  | **ICD-9** | **ICD-10** | **Self-reported fields** |
| **Inflammatory bowel disease** | 556, 5569, 555, 5550,  5551, 5552, 5559, | K51, K510, K511, K512,  K513, K515, K518, K519,  K50, K500, K501, K508,  K509, K514, | 1461, 1462, 1463, |
| **Crohn's disease** | 555, 5550, 5551, 5552,  5559, | K50, K500, K501, K508,  K509, | 1462, |
| **Ulcerative colitis** | 556, 5569, | K51, K510, K511, K512,  K513, K515, K518, K519,  K514, | 1463, |

| **Supplementary Table 2. Diet component definitions used in the UK Biobank study** | | | |
| --- | --- | --- | --- |
| **Components** | **Intake goal** | **Field IDs** | **Amount per serving** |
| Fruit | 3 servings/day | 1309 (pieces fresh fruit/day)  1319 (pieces dried fruit/day) | 1309 – 1 piece 1319 – 5 pieces |
| Vegetable | 3 servings/day | 1289 (tablespoons cooked vegetables/day)  1299 (salad/raw vegetables/day) | 3 heaped tablespoons |
| Whole grains | 3 servings/day | 1438, 1448 (wholemeal/wholegrain bread slices/week) 1458, 1468 (bran/oat/muesli cereal bowls/week) | 1438/1448 – 1 slice/day  1458/1468 – 1 bowl/day |
| Fish | ≥2 servings/week | 1329 (oily fish/week) 1339 (non-oily fish/week) | Once/week |
| Dairy | 2 servings/day | 1408 (cheese/week) 1418 (milk type) | 1408 – 1 piece/day 1418 – 1 glass/day if consumption of any type of milk |
| Vegetable oils | 2 servings/day | 1428 (Flora Pro-Active/Benecol spread) 2654 (Flora Pro-Active/Benecol, soft margarine -, olive oil based -, polyunsaturated/sunflower oil based -, other low/reduced fat spread) 1438 (bread slices/week) | 1 serving/day if in combination with eating at least 2 slices of bread (ID 1438) |
| Refined grains | ≤2 servings/day | 1438, 1448 (white, brown, other bread slices/week)  1458, 1468 (biscuit, other cereals/week) | 1438/1448 – 1 slice/day  1458/1468 – 1 bowl/day |
| Processed meats | ≤1 serving/week | 1349 (processed meat/week or daily)  3680 (age when last ate meat) | 1349 – 1 piece/day 3680 – 0 pieces/day if indicated having never eaten meat |
| Unprocessed meats | ≤2 serving/week | 1359 (poultry/week or day)  1369 (beef/week or day) 1379 (lamb or mutton/week or day)  1389 (pork/week or day) 3680 (age when last ate meat) | 1359-1389 – once/week 3680 – 0 pieces/day if indicated having never eaten meat |
| Sugar-sweetened beverages | Don’t drink | 6144 (never consumes drinks containing sugar) | 0 servings |
| *Field IDs and serving sizes used per diet component in UK Biobank with available data from the general baseline questionnaire. If participants achieved*  *the intake goal they were given 1 point for the diet component. The total healthy diet score was the sum of all the diet component scores and ranged from*  *0 to 10.* | | | |

| **Supplementary Table 3. Spearman correlations between the fish oil use assessed at baseline and in resurveys** | | | |
| --- | --- | --- | --- |
|  | **Baseline** | **First repeat assessment**  **(2012-2013)** | **Imaging visit (2014+)** |
| **No of participants** | 447,890 | 18,093 | 44,366 |
| **Baseline** | 1.00 | 0.61 | 0.47 |
| **First repeat assessment (2012-13)** |  | 1.00 | 0.62 |
| **Imaging visit (2014+)** |  |  | 1.00 |

| **Supplementary Table 4 HRs (95% CIs) of inflammatory bowel diseases in sensitivity**  **analysis** | | | | |  |  |  |  |
| --- | --- | --- | --- | --- | --- | --- | --- | --- |
|  | **Fish oil-users** | **Fish oil users** | **P values** |  |  |  |  |  |
| **Excluding incident IBD cases within 2 years** |  |  |  |  |  |  |  |  |
| Number of participants | 307,876 | 139,682 |  |  |  |  |  |  |
| Number of case (%) | 908 (0.29) | 406 (0.29) |  |  |  |  |  |  |
| Age- and sex-adjusted HR (95% CI) | 1[Reference] | 0.94 (0.83-1.05) | .27 |  |  |  |  |  |
| MV- adjusted HR (95% CI) ^a^ | 1[Reference] | 0.88 (0.77-1.00) | **.04** |  |  |  |  |  |
| **Excluding extreme BMIs** |  |  |  |  |  |  |  |  |
| Number of participants | 298,318 | 136,394 |  |  |  |  |  |  |
| Number of case (%) | 1,106 (0.37) | 487 (0.36) |  |  |  |  |  |  |
| Age- and sex-adjusted HR (95% CI) | 1[Reference] | 0.93 (0.83-1.03) | .16 |  |  |  |  |  |
| MV- adjusted HR (95% CI) | 1[Reference] | 0.88 (0.78-0.99) | **.03** |  |  |  |  |  |
| **Further adjustment for coffee intake** |  |  |  |  |  |  |  |  |
| Number of participants | 308,111 | 139,779 |  |  |  |  |  |  |
| Number of case (%) | 1,143 (0.37) | 503 (0.36) |  |  |  |  |  |  |
| Age- and sex-adjusted HR (95% CI) | 1[Reference] | 0.93 (0.83-1.03) | .16 |  |  |  |  |  |
| MV- adjusted HR (95% CI) | 1[Reference] | 0.88 (0.78-0.99) | **.03** |  |  |  |  |  |
| **Further adjustment for contraceptive** |  |  |  |  |  |  |  |  |
| Number of participants | 308,111 | 139,779 |  |  |  |  |  |  |
| Number of case (%) | 1,143 (0.37) | 503 (0.36) |  |  |  |  |  |  |
| Age- and sex-adjusted HRs (95% CI) | 1[Reference] | 0.93 (0.83-1.03) | .16 |  |  |  |  |  |
| MV- adjusted HR (95% CI) | 1[Reference] | 0.88 (0.78-0.99) | **.03** |  |  |  |  |  |
| **Further adjusted for depression** |  |  |  |  |  |  |  |  |
| Number of participants | 308,111 | 139,779 |  |  |  |  |  |  |
| Number of case (%) | 1,143 (0.37) | 503 (0.36) |  |  |  |  |  |  |
| Age- and sex-adjusted HR (95% CI) | 1[Reference] | 0.93 (0.83-1.03) | .17 |  |  |  |  |  |
| MV- adjusted HR (95% CI) | 1[Reference] | 0.88 (0.78-0.99) | **.03** |  |  |  |  |  |
| **Further adjusted for C-reactive protein** |  |  |  |  |  |  |  |  |
| Number of participants | 308,111 | 139,779 |  |  |  |  |  |  |
| Number of case (%) | 1,143 (0.37) | 503 (0.36) |  |  |  |  |  |  |
| Age- and sex-adjusted HR (95% CI) | 1[Reference] | 0.94 (0.84-1.05) | .25 |  |  |  |  |  |
| MV- adjusted HR (95% CI) | 1[Reference] | 0.89 (0.79-0.99) | **.04** |  |  |  |  |  |
| **Further adjusted for albumin** |  |  |  |  |  |  |  |  |
| Number of participants | 30,8111 | 139,779 |  |  |  |  |  |  |
| Number of case (%) | 1,143 (0.37) | 503 (0.36) |  |  |  |  |  |  |
| Age- and sex-adjusted HR (95% CI) | 1[Reference] | 0.93 (0.83-1.03) | .17 |  |  |  |  |  |
| MV- adjusted HR (95% CI) | 1[Reference] | 0.88 (0.79-0.99) | **.03** |  |  |  |  |  |
| *CIs - confidence intervals; HRs - hazard ratios; MV - multivariable; Contraceptive - oral contraceptive pill or minipill;*  *^a^ Models was adjusted for age, sex, race (White, Asian, Black, mixed, or other ethnic group), assessment centers (22 categories), BMI (in kg/m^2^; <18.5, 18.5 to 25, 25 to 30, 30 to 35, ≥35, or missing), education (college or university degree, vocational qualifications, optional national exams at ages 17–18 years, national exams at age 16 years, others, or missing), Townsend deprivation index (quintiles), household income (<£18,000, £18,000-£30,999, £31,000-£51,999, £52,000-£100,000, >£100,000, or missing), smoking status (never, former, current, or missing), alcohol consumption (never, special occasions only, 1 to 3 times/month, 1 or 2 times/week, 3 or 4 times/week, or daily/almost daily), physical activity (in MET-h/wk; quintiles), vitamin supplement use (yes or no), mineral supplement use (yes or no), aspirin use (yes or no), Hormone use (yes or no), nonsteroidal anti-inflammatory drugs use (yes or no), healthy diet score (quintiles). Further analysis included excluding incident IBD cases within 2 years, extreme BMI (<18.5 or >40 kg/m2), coffee intake (0-1 cup/day, 1-3 cup/day, ≥3 cup/day), contraceptive (yes or no), depression (yes or no), C-reactive protein (<5mg/l, 5-10mg/l, ≥10mg/l), albumin (<35g/l, 35-50g/l, ≥50g/l).* | | | |  |  |  |  |  |
|  |  |  |  |  |  |  |  |  |
|  |  |  |  |  |  |  |  |  |
